# Combined renal and common hepatic artery denervation as a novel approach to reduce cardiometabolic risk: technical approach, feasibility and safety in a pre-clinical model

**DOI:** 10.1007/s00392-021-01814-1

**Published:** 2021-02-26

**Authors:** Márcio Galindo Kiuchi, Kavitha Ganesan, John Keating, Revathy Carnagarin, Vance B. Matthews, Lakshini Y. Herat, Gerard Goh, Leon Adams, Markus P. Schlaich

**Affiliations:** 1 Dobney Hypertension Centre, Faculty of Medicine, School of Medicine-Royal Perth Hospital Unit, Dentistry and Health Sciences, The University of Western Australia, Level 3, MRF Building, Rear 50 Murray St, Perth, WA 6000 Australia; 2 Metavention Inc, Maple Grove, MN USA; 3 CBSET, Inc, Lexington, MA USA; 4 Radiology Department, Department of Surgery, Central Clinical School Alfred Hospital, Monash University, Melbourne, VIC Australia; 5 Medical School, The University of Western Australia, Perth, WA Australia; 6 Neurovascular Hypertension and Kidney Disease Laboratories, Baker Heart and Diabetes Institute, Melbourne, Australia; 7 Departments of Cardiology and Nephrology, Royal Perth Hospital, Perth, Australia

**Keywords:** Sympathetic nervous system, Renal denervation, Hepatic denervation, Cardiometabolic risk, Hypertension, iRF denervation

## Abstract

**Background:**

Cardiovascular and metabolic regulation is governed by neurohumoral signalling in relevant organs such as kidney, liver, pancreas, duodenum, adipose tissue, and skeletal muscle. Combined targeting of relevant neural outflows may provide a unique therapeutic opportunity for cardiometabolic disease.

**Objectives:**

We aimed to investigate the feasibility, safety, and performance of a novel device-based approach for multi-organ denervation in a swine model over 30 and 90 days of follow-up.

**Methods:**

Five Yorkshire cross pigs underwent combined percutaneous denervation in the renal arteries and the common hepatic artery (CHA) with the iRF Denervation System. Control animals (*n* = 3) were also studied. Specific energy doses were administered in the renal arteries and CHA. Blood was collected at 30 and 90 days. All animals had a pre-terminal procedure angiography. Tissue samples were collected for norepinephrine (NEPI) bioanalysis. Histopathological evaluation of collateral structures and tissues near the treatment sites was performed to assess treatment safety.

**Results:**

All animals entered and exited the study in good health. No stenosis or vessel abnormalities were present. No significant changes in serum chemistry occurred. NEPI concentrations were significantly reduced in the liver (− 88%, *p* = 0.005), kidneys (− 78%, *p* < 0.001), pancreas (− 78%, *p* = 0.018) and duodenum (− 95%, *p* = 0.028) following multi-organ denervation treatment compared to control animals. Histologic findings were consistent with favourable tissue responses at 90 days follow-up.

**Conclusions:**

Significant and sustained denervation of the treated organs was achieved at 90 days without major safety events. Our findings demonstrate the feasibility of multi-organ denervation using a novel iRF Denervation System in a single procedure.

## Introduction

Cardiovascular and metabolic regulation is governed by neurohumoral signalling from the sympathetic nervous system (SNS) in relevant organs such as kidney, liver, pancreas, skeletal muscle and adipose tissue. Increased sympathetic tone is a hallmark of essential hypertension [1–4] and plays an important role in its pathogenesis. Sympathetic activation has also been associated with increased cardiovascular risk [5, 6]. Aside from its role in cardiovascular control, activation of the SNS alters insulin resistance [7] and is implicated in metabolic syndrome [8], central obesity [9], and the development of type 2 diabetes mellitus (T2DM) [10]. Hypertension and T2DM commonly coexist, thereby further increasing the risk of developing cardiovascular complications [11–13].

Currently, efficient management and treatment of hypertension and T2DM require concerted efforts from both physicians and patients to combine effective non-pharmacological and pharmacological approaches to prevent hypertensive target organ damage and diabetic macro and micro-vascular complications [14–17]. Adherence to antihypertensive medication is highly variable in clinical trials [18, 19], and non-adherence has been shown in up to 60% of patients in some studies [20]. Similarly, in patients with T2DM, non-adherence to antidiabetic drugs may range from 53 to 65% and may be responsible for uncontrolled glucose levels in about 23% of cases [21]. In view of these data, it appears appropriate to explore novel therapeutic strategies that may rely less on daily adherence with prescribed medications.

One such alternative approach extensively trialled over the last decade is catheter-based renal denervation (RDN). This minimally invasive procedure has been reported to be safe and effective in reducing blood pressure in a variety of relevant patient cohorts [20, 22–25]. Doubts regarding its efficacy have been put aside by four recent sham-controlled randomized controlled trials [20, 22–25].

Of importance in the current context and complementing the proof of safety and BP lowering efficacy of catheter-based RDN in sham-controlled trials [20, 22–25], experimental studies have demonstrated an important role of the SNS in the regulation of glucose metabolism during the development of type 2 diabetes [26, 27]. Although two clinical studies have shown neutral effects of RDN on insulin and glucose levels [28, 29], ancillary effects of RDN in relation to glucose metabolism and insulin sensitivity in hypertensive patients [30–33] have been reported.

Furthermore, both experimental and clinical evidence indicates that sympathetic tone to the liver, intestine, pancreas and peripheral tissue can be a critical modulator of glucose production, insulin secretion, peripheral glucose uptake and that increased central sympathetic signalling to these key organs contributes to the development of T2DM [27, 34, 35].

Taken together, these data suggest that the intricate interplay between afferent and efferent signalling from organs relevant to both cardiovascular and metabolic control such as the kidney, liver, and pancreas may provide a unique therapeutic opportunity to selectively interrupt crucial neural signalling pathways to improve both cardiovascular and metabolic alterations. In fact, cardiometabolic neuromodulation via multi-organ sympathetic denervation could potentially provide a holistic approach to combat the cluster of metabolic abnormalities frequently encountered in patients at increased cardio-metabolic risk including those with obesity, metabolic syndrome, hypertension and T2DM.

Against this background, we report here on the technical approach of a novel multi-electrode RF denervation system in a preclinical large animal model. We aimed to investigate the feasibility, safety, and performance of the device for multi-organ denervation in a swine model over 30 and 90 days of follow-up.

## Methods

### Device overview

**Fig. 1:**
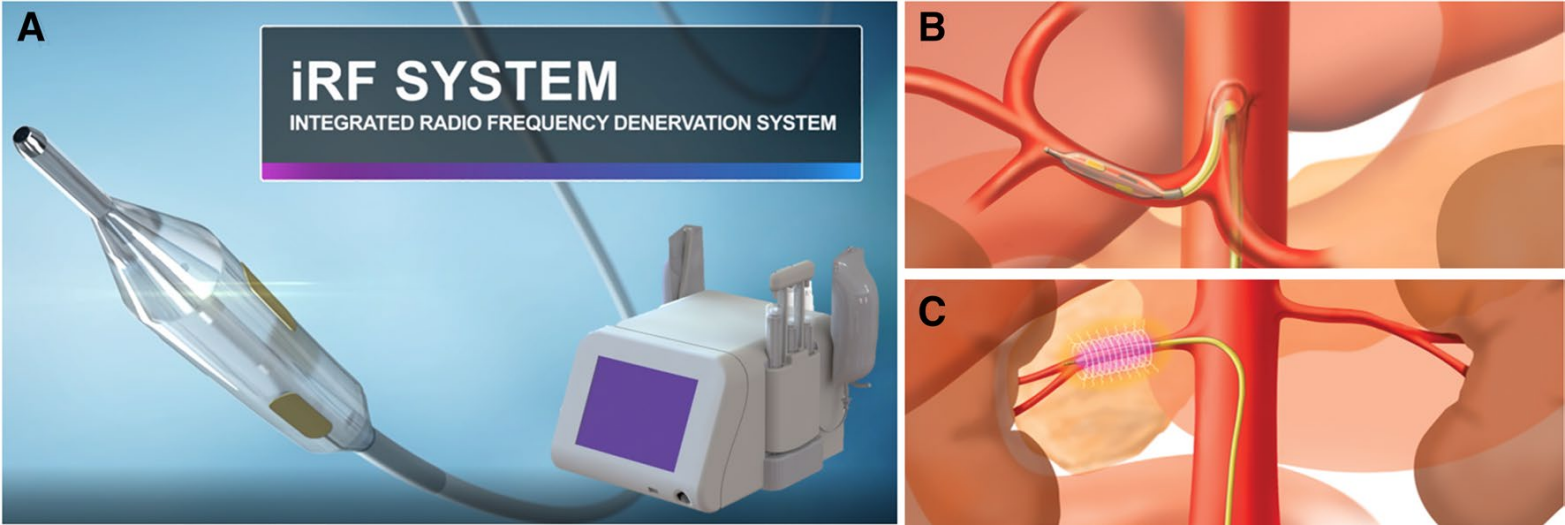
Integrated radiofrequency (iRF) denervation system. **a** Multi-electrode RF denervation catheter and generator. **b** Illustration of the catheter positioned within the common hepatic artery. **c** Illustration of circumferential heating during RF delivery in the renal artery

The iRF Denervation System (Metavention, Maple Grove, MN, USA) is a second generation percutaneous, catheter-based device which uses RF energy to circumferentially denervate the sympathetic nerves surrounding the renal arteries and the CHA. The iRF system (Fig. 1) consists of a multi-electrode catheter assembly which is designed to produce a long blended circumferential lesion with a single treatment to denervate the perivascular space surrounding the arteries. The iRF catheter is a standard over the wire (OTW) balloon design which provides uniform wall engagement and electrode tissue contact for optimal electrode energy transfer and minimize patient to patient or user to user variabilities. Tissue heating results when current passes through the electrically resistive tissue. Power density in the tissue is highest near the electrode and decreases rapidly with distance. The power density on the electrode surface is offset by the iRF system’s active internal cooling system thereby preserving the integrity of the arterial intima and media while creating desired tissue heating in the adventitial space.

### Animal model

To support the use of the porcine model for the evaluation of the iRF Denervation System, a preclinical study was conducted in ten swine aiming to distinguish the type and location of the nerves and structures within the perivascular space surrounding the CHA [36]. Results revealed > 95% of the nerves surrounding the CHA were efferent sympathetic nerves. Lymph nodes, pancreas and adjacent vessels (i.e., portal vein) were identified as the most common tissue type surrounding the CHA. Lymph nodes and nearby vessels were, on average, 2.3 and 2.0 mm from the CHA lumen. The pancreas was, on average, 2.4 mm from the CHA lumen with minimal distances ranging from 0.9 mm to 7.6 mm [36]. The type of perivascular tissue and proximity have been previously studied in the renal arteries [37, 38, 42] and are similar [39–41] with adjacent vessels (1–2 mm), lymph nodes (2 mm), organs such as bowel (3.5–7 mm), pancreas (0.9–7.6 mm), liver (4.3–7.8 mm) and muscle (3–6 mm).

A detailed histological evaluation and characterization of the differences between the CHA nerve distribution and surrounding tissues for porcine and human cadavers was performed to further understand and support the use of the porcine model (report on file with Metavention). The results of this evaluation indicate that porcine CHA nerves are representative of human anatomy. Notably, the nerves and tissues of interest (pancreas, lymph nodes and vessels) are closer to the CHA luminal surface in the porcine model. Therefore, the porcine model represents a conservative (i.e., stringent) safety model for human therapy evaluation because (1) depths will extend further in the animal anatomy to approximate the greater observed depth of nerves in the human, and (2) the probability of non-targeted tissues (i.e., pancreas, bowel, psoas muscle) residing in the thermal denervation zone is higher in the animal model. Collectively, these findings support the use of the porcine model for both evaluation of the iRF Denervation System [36].

### Preclinical study design

The iRF system was evaluated in porcine models to confirm the feasibility, safety, and effectiveness of the iRF Denervation System for multi-organ denervation. The studies were conducted at American Preclinical Services (Minneapolis, MN, USA), an Association for Assessment and Accreditation of Laboratory Animal Care (AAALAC) accredited test facility. The protocol for these studies was reviewed and approved by the Institutional Animal Care and Use Committee (IACUC) and complied with the USDA Regulations and the Animal Welfare Act.

Anaesthesia was induced with multiple agents. A Dissociative anaesthetic, Tiletamine/Zolazepam, 3.5–5.5 mg/kg/Xylazine, 1.5–3.5 mg/kg was delivered intramuscular to start the induction. Propofol, typical dose 2–8 mg/kg, was then given intravenously in addition to a gas anaesthetic, Isoflurane in 100% O_2_, 0–5%, inhale, to effect to complete the induction. The gas anaesthetic was used to maintain surgical plane of anaesthesia during the procedure per institution protocols.

Five Yorkshire cross pigs underwent percutaneous denervation in the renal arteries and the CHA with the iRF Denervation System with follow-up at 90 days. Animals were allowed up to two treatments, provided the length of the arteries could accommodate two non-overlapping treatments. In the control group, three Yorkshire cross pigs were included in the study. The control group was used to provide baseline reference data on animals that underwent an equivalent intravascular procedure without introduction of the iRF catheter or energy delivery to the vessel wall. Treatment and control animals were randomly assigned and were of similar age and weight.

**Fig. 2:**
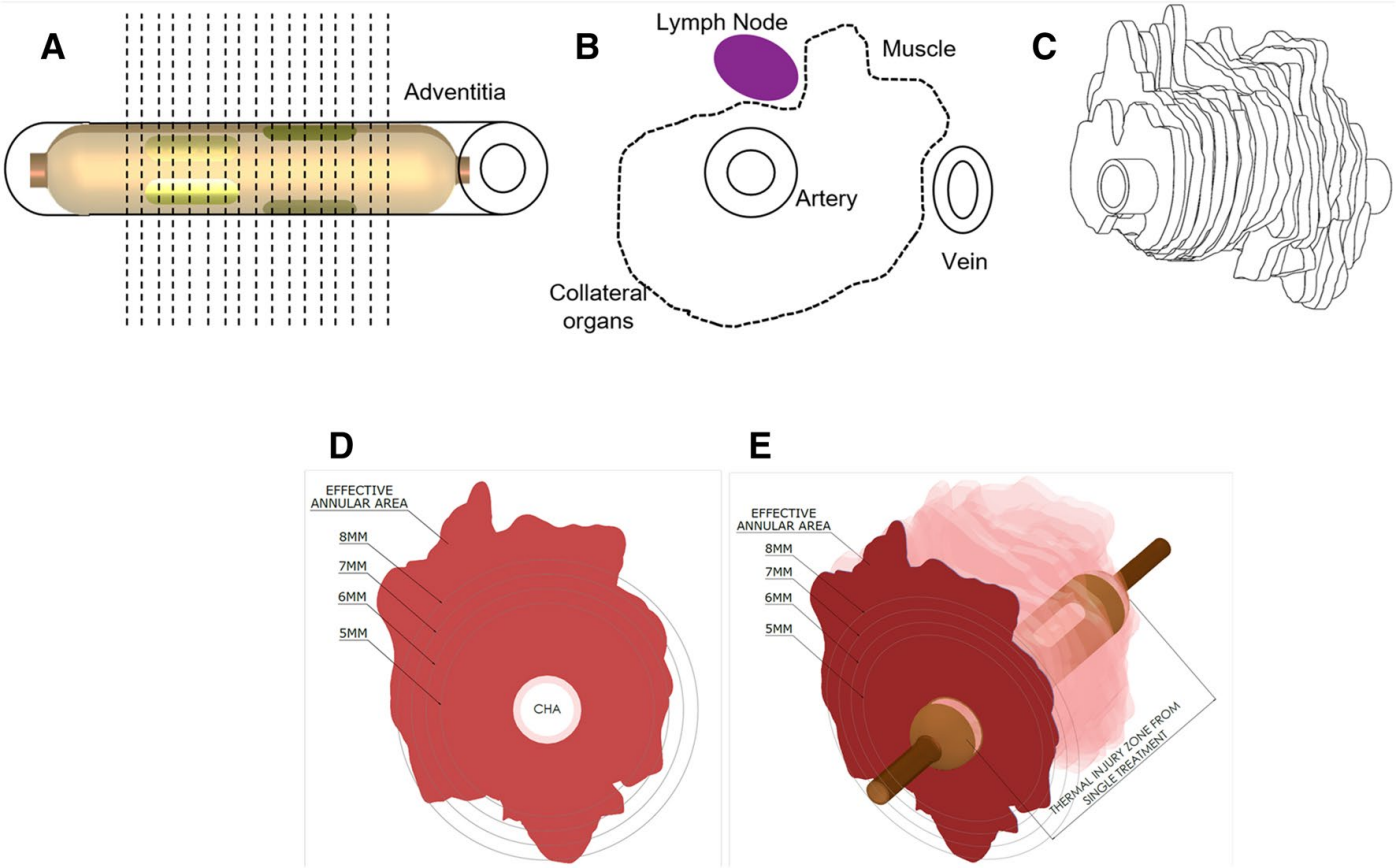
Annularity model, **a** fine sectioning method (1,000 umincrements) used to produce several sections from each treatment artery. **b** Thermal injury area tracing to assess the circumferenciality of treatment from each section. **c** Virtual treatment model generated by cumulative overlay of thermal injury tracings from each step section. **d**, **e** Virtual annularity model showing effective annular area from a single CHA treatment

Specific energy doses (power and time) were administered in the renal arteries and CHA which were previously characterized in 7-day dosing studies (report on file with Metavention). Histomorphology of thermal injury at 7 days post-treatment was traced using Aperio Image Scope software on high resolution scanned slide images by an independent board-certified pathologist (Rippy Pathology Solutions). The trace on each slide was used to cumulatively build a 3-dimensional Annularity Model (Fig. 2a–c) of the entire treatment zone. These models (Fig. 2d, e) estimated a % effective thermal injury area surrounding the treatment artery to various depths (e.g., 6 mm, 7 mm, 8 mm, etc.). The 5 W 120 s dose demonstrated a 90% average treatment depth within 6 mm, and the 6 W 150 s animals showed an 88% average treatment depth close to 8 mm (Table 1). These data allowed comparison and selection of the appropriate energy doses for renal and CHA treatments to achieve the depth target while minimizing treatment effects on the vessel and non-targeted tissues surrounding each treatment location. A fixed electrode cooling flow rate was used for all animals to further protect the artery vessel wall from thermal injury.

**Table 1:** Dose summary

Treatment		% Effective thermal injury area		
	Within 6 mm	Within 7 mm	Within 8 mm	> 8 mm
Renal dose (5 W 120S)	90 ± 3	81 ± 13	72 ± 16	15 ± 13
CHA dose (6 W 150S)	99 ± 1	94 ± 6	88 ± 8	28 ± 21

### Multi-organ denervation procedure

All animals were treated with aspirin (325 mg PO) and clopidogrel (150 mg PO) 24 h prior to the procedure. Following general anaesthesia, a 9 Fr introducer sheath was placed via the femoral artery, and intravenous heparin administered to achieve an activated clotting time (ACT) of ≥ 250seconds. Angiography of the renal arteries and CHA were performed prior to energy delivery (baseline images). For all treatment animals, vessel diameter and length measurements were obtained using quantitative vascular analysis (QVA) to determine the appropriate catheter size and number of planned treatments for each treatment artery, i.e. the CHA, left renal artery (LRA) and right renal artery (RRA). An 8 Fr guide catheter or 7 F sheath was placed in the celiac axis just proximal to the CHA/splenic bifurcation. The chosen iRF catheter was then introduced over a 0.014″ guidewire via fluoroscopic guidance and advanced to the distal most section of the CHA. Device position and proper apposition of the balloon was confirmed using a contrast injection prior to starting the treatment cycle. Radiofrequency (RF) energy was then delivered to the CHA. Following completion of energy delivery at the distal location, if the artery length was adequate the balloon was deflated, and the catheter repositioned proximally for a second treatment. Once CHA treatments were complete, a post-procedure angiogram was performed, and any severe spasm treated with Nitroglycerin. A new iRF catheter was used to perform renal artery treatments following the same procedure above. Both left and right renal arteries were treated. Similar to the CHA treatment, the iRF catheter was positioned as far distal as feasible within the main renal artery prior to treatment. Distal branches and any accessories if present were left untreated. Post-procedure, all animals were treated with aspirin (81 mg PO) and clopidogrel (75 mg PO) daily for 7 days. The animals were survived until their scheduled termination at 90 days. Euthanasia was performed via lethal injection with a euthanasia solution per institutional protocols administered at ~ 1 ml per 4.5 kg dose. The euthanasia solution was composed of pentobarbital sodium 390 mg/ml, propylene glycol 0.01 mg/ml, ethyl alcohol 0.29 mg/ml, and benzyl alcohol (preservative) 0.2 mg/ml. Death was verified by auscultation or pulse monitoring.

### Follow-up procedures

#### Animal health and clinical pathology

Overall animal health was monitored throughout the study period including daily clinical observations and physical exams by the study veterinarian. Blood was collected for clinical pathology analyses at a 30-day interim time point and at a pre-term time point of 90 days. Clinical pathology included, standard hematology (CBC) panel and a custom serum chemistry panel which included urea nitrogen (BUN), creatinine, total protein, albumin, aspartate aminotransferase (AST), lactate dehydrogenase (LDH), alanine aminotransferase (ALT), alkaline phosphatase (ALP), gamma GT (GGT), total bilirubin, glutamate dehydrogenase (GLDH), amylase, lipase creatine kinase (CK), cholesterol, triglycerides bicarbonate, glucose, sodium, potassium, chloride, calcium, and phosphorus.

#### Angiography

All animals, treatment and control went through a pre-terminal procedure angiography of the treated arteries CHA, RRA, and LRA. QVA was performed to determine vessel diameters at 90 days. The % narrowing or stenosis, defined as > 50% narrowing due to treatment was estimated by an independent study radiologist.

#### Tissue collection and bioanalysis

Prior to euthanasia and necropsy tissue procurement, a laparotomy was performed under sedation to collect tissue samples for norepinephrine (NEPI) bioanalysis. Gross examination of collateral tissue near the treatment sites (bowels, GI tract, duodenum, liver, pancreas) was conducted prior to collection of 1 g tissue form each kidney (cortex), liver lobe, pancreas, and duodenum. The tissues were flash frozen and sent for NEPI analysis to an independent laboratory for analysis (Vanderbilt University Medical Center Hormone Assay and Analytical Services Core, Nashville, Tennessee). Animals were then euthanized and sent to necropsy for a complete gross pathology observation and histological tissue procurement.

#### Histopathological evaluation

Gross examination was conducted of the heart and all tissues and collateral structures near the treatment sites. This included bowel, liver, pancreas, spleen, kidneys, psoas muscle and the treated vessels. The treated arteries were then perfused with lactated Ringer’s solution followed by 10% neutral buffered formalin (NBF) for in situ fixation. The treatment arteries were then removed “en-bloc” without disturbing associated peri-arterial tissue and organs and immersion fixed in 10% NBF for histology processing and examination. All tissue with a gross finding was also collected for histologic processing and examination. Histology slides were shipped to CBSET, Inc. for histological assessment by a board-certified veterinary pathologist. Injury assessment to vessel wall and perivascular tissue surrounding each treatment site was used to assess the safety profile of the iRF Denervation System. Chronic treatment effects on both the artery wall and surrounding tissues were assessed on all produced sections under a light microscope and graded semi quantitatively on a scale of 0–4. Mean ± SD, Median scores and % incidence of observations were reported in all animals. Table 2 shows the summary of assessments performed during the multi-organ denervation safety study.

**Table 2:** Safety and performance parameters

Assessment	Purpose	Follow-up timepoints	Evaluator
Angiography	To assess vessel patency	Baseline, procedure, and sacrifice (90 days)	Independent board-certified radiologist
Animal health	To assess animal health for any clinically significant observations	Monitored throughout the study duration through sacrifice (90 days)	Independent board-certified veterinarian
Clinical pathology (bloodwork)	To assess hematology and serum chemistry values related to animal health, including: • Liver • Pancreas • Kidney function	Pre-treatment, intermediate time point (30 days) and at sacrifice (90 days)	Independent board-certified veterinarian
Gross pathology (necropsy)	To assess any gross abnormalities, including: • Hepatic parenchyma of all liver lobes • Head, body, and tail of the pancreas • Proximal duodenum • Gall bladder • Kidneys • Small bowel • Psoas muscle adjacent to the renal arteries	At sacrifice (90 days)	Independent board-certified veterinary pathologist
Histopathology of the treatment area (renal arteries, common hepatic artery and surrounding tissues)	To assess: • Morphologic characterization of treatment area, including depth and circumferentiality • Vessel wall injury • Perivascular injury	At sacrifice (90 days)	Independent board-certified veterinary pathologist
Norepinephrine assessment	To assess denervation of the liver, kidneys and adjacent organs (pancreas and duodenum)	At sacrifice (90 days)	Independent test laboratory

### Statistical analysis

Data are presented as mean ± standard deviation. A one-way analysis of variance (ANOVA) was used to examine differences in various enzymes and markers from baseline to terminal across the groups. NEPI concentration from liver, kidney, pancreas and duodenum from treated group was compared with those from control group using a Two-sample *t* test assuming unequal variances. A *p* value < 0.05 was considered to indicate statistical significance.

## Results

### Multi-organ safety study findings

The iRF Denervation System performed as intended with successful navigation of the iRF catheter to the bilateral renal arteries and CHA locations in all five treatment animals. The delivery of the intended energy dose(s) to all treatment arteries was completed per protocol.

All animals survived the treatment period including the controls with no safety concerns. All animals entered and exited the study in good health. All animals gained weight appropriately throughout the study duration. No stenosis or vessel abnormalities were present in renal arteries and CHA. Gross and microscopic pathology results demonstrate the iRF Denervation System met the pathology objective of this study.

The relevant outcomes assessed during the multi-organ denervation safety study are summarized in Table 3.

**Table 3:** Safety summary

Assessment	Summary
Angiography	No stenoses present at 90 days
Animal health	All animals were healthy, and no clinically significant changes were noted throughout the duration of the study
Clinical pathology (bloodwork)	No clinically significant observations or abnormal trending was observed between baseline, interim (30 days) and terminal (90 days) timepoints for treated vs. control animals
Gross pathology (necropsy)	No macroscopic evidence of injury to downstream or adjacent tissues (small bowel, psoas muscle, kidneys, liver, gall bladder, proximal duodenum, and head, body and tail of the pancreas)
Histopathology of the treatment area (common hepatic artery and surrounding tissues)	Histologic findings were consistent with favourable tissue responses 90 days following treatment: • Treated vessels were completely endothelialized and did not contain any evidence of thrombus • There was no evidence of clinically significant mural injury (e.g., arterial dissection), excessive neointima formation or occlusive process • Repair and remodelling of vessel walls and perivascular tissue was considered complete • Inflammation was minimal to absent
Norepinephrine assessment	Average NE reductions at 90 days for the treated animals vs. controls Liver: 88% Pancreas: 78% Duodenum: 95% Kidney: 78%

No clinically significant observations were made after examining the animal health and clinical pathology values for both the treated and control animals. Change in serum chemistry specific to liver, pancreas and kidney function are summarized in Table 4 showing no significant change between baseline to 30- and 90-day follow-up time points. Although the study was conducted in healthy swine, glucose and lipid (cholestrerol and tryglycerides) levels were monitored at 30 and 90 days (Table 4).

**Table 4:** Change in serum chemistry between baseline and each time point (30 days and 90 days)

Clinical pathology parameter	Δ (Baseline to 30 days)			Δ (Baseline to 90 days)		
	Treatment	Control	*p*-value	Treatment	Control	*p*-value
Aspartate aminotransferaces test (AST)	26.8 ± 38.41	2.0 ± 5.29	0.323	2.8 ± 11.5	16.33 ± 7.51	0.123
Alanine aminotransferase test (ALT)	15.6 ± 14.3	13.0 ± 9.0	0.790	20.4 ± 14.01	26.33 ± 14.74	0.589
Alkaline phosphatase test (ALP)	− 2.0 ± 49.89	8.33 ± 25.06	0.796	− 36.8 ± 54.36	− 45.7 ± 36.02	0.813
Gamma-glutamyl transferase (GGT)	0.40 ± 7.29	4.53 ± 4.12	0.412	− 3.64 ± 5.87	− 0.74 ± 2.07	0.451
Creatinine	0.02 ± 0.18	0.03 ± 0.12	0.913	0.16 ± 0.21	0.23 ± 0.31	0.696
Albumin	− 0.08 ± 0.16	− 0.1 ± 0.1	0.857	− 0.06 ± 0.3	0 ± 0.26	0.784
Albumin globulin (A/G) ratio	0.08 ± 0.13	− 0.03 ± 0.12	0.263	− 0.22 ± 0.19	− 0.43 ± 0.12	0.138
Amylase (VAMY)	457 ± 613.95	342 ± 462.52	0.791	508 ± 509	675 ± 357.79	0.640
Lipase (LIPASE)	9.0 ± 36.24	− 3.0 ± 3.61	0.600	7.4 ± 9.29	10 ± 13.89	0.758
Glucose	− 21.0 ± 27.63	− 23.67 ± 22.37	0.893	− 14.0 ± 18.96	− 8.67 ± 22.37	0.730
Cholesterol	− 2.8 ± 14.10	− 11.00 ± 13.86	0.454	− 4.2 ± 9.83	2.0 ± 13.86	0.482
Tryglycerides	12.42 ± 9.56	23.73 ± 25.97	0.395	− 3.94 ± 4.45	− 4.13 ± 5.63	0.958

One-way analysis of variance (ANOVA) was used to examine differences in serum chemistry between treatment and control groups at each end points

A *p* value greater than 0.05 was considered insignificant

**Fig. 3:**
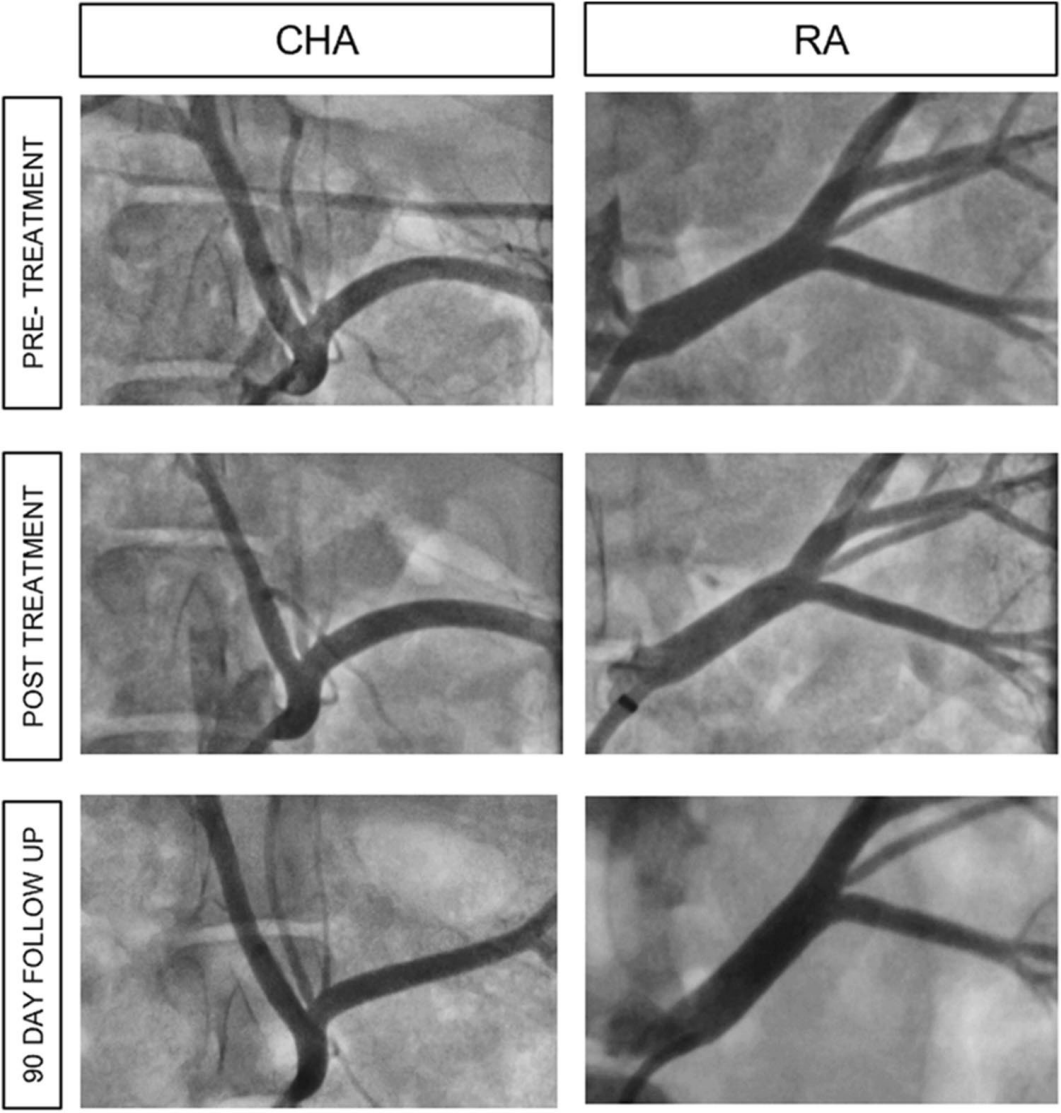
Angiography. Representative angiographic images of CHA and RA at pre-treatment, post-treatment, and follow-up (90 days). Immediately after RF treatment, occasional spasm was noted, while no other abnormalities such as dissection or perforation were found. Follow-up angiography indicated no narrowing of lumen or signs of stenosis, thrombus, or other abnormalities at 90 days

No narrowing or stenosis was observed in any of the treated or control animals at 90 days post-procedure. No thrombosis, filling defects or other abnormalities were noted (Fig. 3).

Gross pathology assessment showed no macroscopic evidence of clinically significant injury to surrounding structures (small bowel, psoas muscle, renal vein, inferior vena cava, portal vein, pancreas, lymph nodes and bile duct) at 90 days post-treatment.

Histologic findings were consistent with favourable tissue responses at 90 days following treatment.

**Fig. 4:**
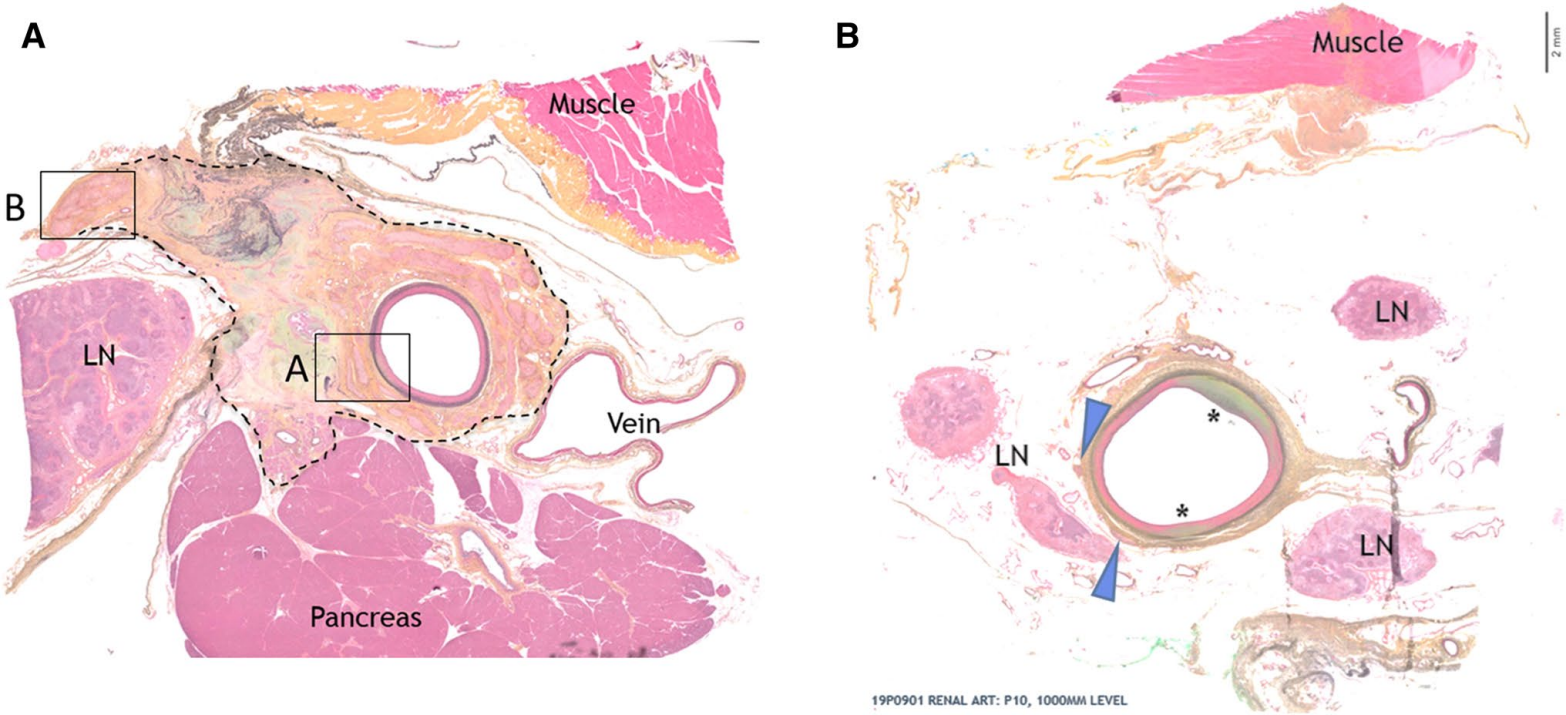
**a** Histology of common hepatic artery, Day 90, Movat's Pentachrome stain. Dashed line indicates extent of chronic adventitial/peri-arterial fibrotic response to RF treatment, encompassing many large nerve bundles that are partially obscured by treatment. There is focal fibrosis/atrophy of adjacent pancreas but no associated necrosis/ inflammation. Boxes A and B are shown at higher magnification in Fig. 5a, b. **b** Histology of renal artery, Day 90, Movat's Pentachrome stain. This section of the left RA contains two areas of vessel response (~ 50% circumference) with minimal annular perivascular fibrosis. Perivascular nerves (arrow heads) are very sparse, suggestive of treatment-related effacement of other nerves segments. Adjacent lymph nodes and veins are unaffected

**Fig. 5:**
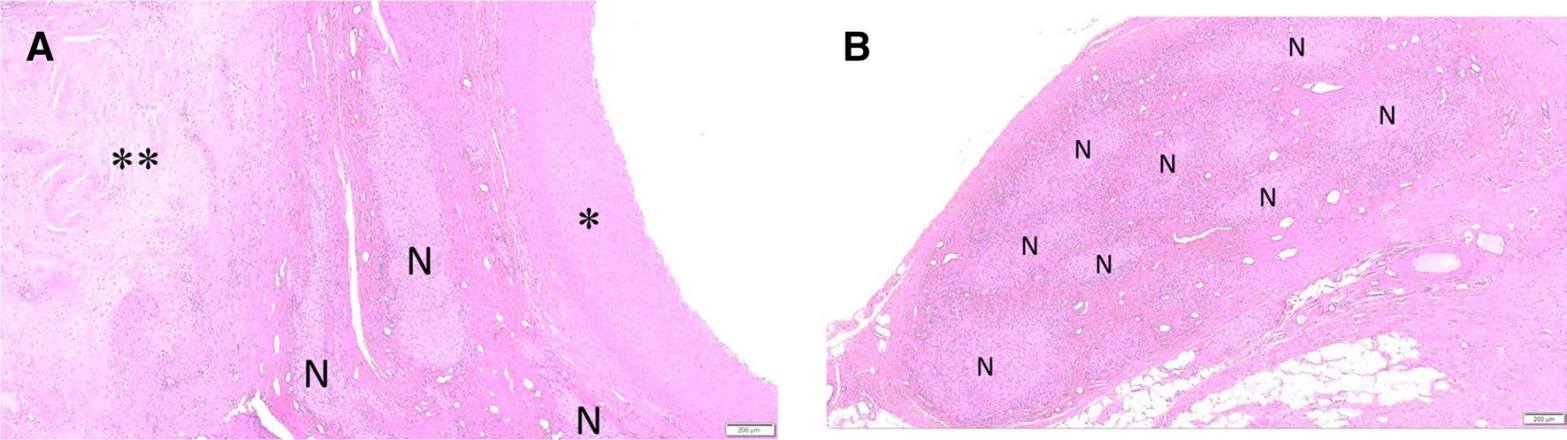
Histology of nerve injury, H and E stain. **a** Higher magnification view from Box A in Fig. 4a. The treated arterial wall (asterisk) exhibits faint pallor but no other changes. Adventitial nerves (N) immediately adjacent to the CHA exhibit mild chronic fibrotic and proliferative response to treatment, while there is a larger focus of sclerosis/fibrosis (double asterisk) slightly more distant. **b** Higher magnification view from Box B in Fig. 4a. The large nerve bundle distant from the treated artery exhibits notable chronic fibrotic and proliferative response to treatment, evident within, between and around bundles (*N*) (i.e., endo, peri and epineurial)

Scores from assessment of adjacent tissue are summarized in Table 5. Chronic response to adventitial/perivascular tissue included various levels of annular fibrosis (Fig. 4). Treatment-related responses in adjacent organs (i.e., pancreas, lymph node) were rare and minimal. Nearly all scores were Grade 1–2 (i.e., minimal, or mild) with Grade 3 pancreatic response limited to single animal (Fig. 4a). The pancreatic findings in this animal were presumably related to RF treatment of the CHA but were highly localized and quiescent, with the vast majority of pancreas being within normal limits. One animal exhibited two treatment-related foci of hypaxial muscle fibrosis, one associated with the CHA and one with the LRA. There were no lymph node responses to treatment. RF treatment-associated nerve responses were typical of chronic time point findings and included fibrosis, hyperplasia, atrophy and neuromatous proliferation (Figs. 4b, 5).

**Table 5:** Scores from assessment of relevant tissues adjacent to the treatment area including inflammation, fibrosis, calcification and pancreatic and lymph node response (Mean ± SD, median and incidence) of adjacent tissue assessments

Parameter	Day 90 treated			
	CHA (*n* = 5)		LRA and RRA (*n* = 10)	
Inflammation	0.03 ± 0.08	20%	0.00 ± 0.00	0%
	0.00		0.00	
Fibrosis	1.19 ± 0.69	100%	0.42 ± 0.36	70%
	1.33		0.45	
Calcification	0.24 ± 0.43	40%	0.00 ± 0.00	0%
	0.00		0.00	
Pancreatic response	0.22 ± 0.21	75%	NA ± NA	NA
	0.19		NA	
Lymph node response	0.00 ± 0.00	0%	0.00 ± 0.00	0%
	0.00		0.00	

*CHA* common hepatic artery, *LRA* left renal artery, *RRA* right renal artery, *NA* not applicable

Adjacent tissue assessment: 0 = none; 1 = (minimal) < 20 cells/40 × HDF—changes barely perceptible; 2 = (mild) 21–100 cells/40 × HDF—changes present but involves a small amount of tissue; 3 = (moderate) 101–150 cells/40 × HDF—changes clearly visible and involves a significant proportion of the tissue; 4 = (marked) > 150 cells/40 × HDF—changes prominently visible and involves a major portion of the tissue

**Fig. 6:**
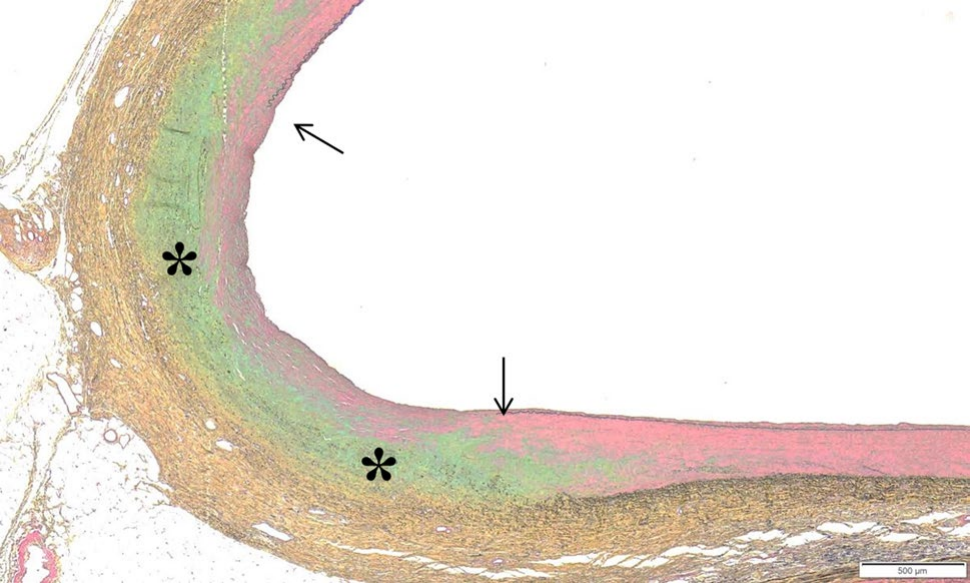
Histology renal artery, Day 90, Movat's Pentachrome stain. Focus of Grade 2 arterial injury (i.e., extent of tear involved tunica media) exhibits reparative adventitial fibrosis (yellow) and medial glycosaminoglycans (green) in the site of injury (asterisks). Arrows indicate edges of torn IEL on either side of the defect

Scores from assessment of vessel wall are summarized in Table 6. There was no evidence of clinically significant mural injury (e.g., arterial dissection), excessive neointima formation or occlusive process in any of the animals. Repair and remodelling of vessel walls and perivascular tissue were considered complete. Inflammation was minimal to absent (Fig. 4a, b). Mechanical injury was absent in the CHA and restricted to renal artery of one animal where a grade 2 injury (i.e., medial laceration) was observed (Fig. 6). The finding was not associated with any adverse events such as vessel occlusion, excessive neointimal proliferation or aneurysm/dissection and considered incidental from otherwise uneventful vascular instrumentation. Luminal thrombus was absent in all animals. There was no evidence of in vivo endothelial cell loss/absence, mural fibrosis was low (mean < 25% of arterial circumference with no individual scores greater than 25–50%) and neointima was nearly absent.

**Table 6:** Scores from assessment of the vessel wall after treatment including injury, fibrosis, endothelilal cell loss, thrombus formation, and neointima formation (Mean ± SD, median and incidence) of vessel wall assessments

Parameter	Day 90 treated			
	CHA (*n* = 5)		LRA and RRA (*n* = 10)	
Injury	0.00 ± 0.00	0%	0.33 ± 0.69	20%
	0.00		0.00	
Fibrosis	0.88 ± 0.28	100%	0.86 ± 0.65	90%
	0.83		0.75	
Endothelial cell loss	0.20 ± 0.45	20%	0.00 ± 0.00	0%
	0.00		0.00	
Thrombus formation	0.00 ± 0.00	0%	0.00 ± 0.00	0%
	0.00		0.00	
Neointima	0.30 ± 0.23	80%	0.40 ± 0.44	50%
	0.33		0.25	

*CHA* common hepatic artery, *LRA* left renal artery, *RRA* right renal artery

Vessel wall assessments: 0 = none; 1 = (minimal) < 25% of the circumference; 2 = (mild) 25–50% of the circumference; 3 = (moderate) 51–75% of the circumference; 4 =  > 75% of the circumference

**Fig. 7:**
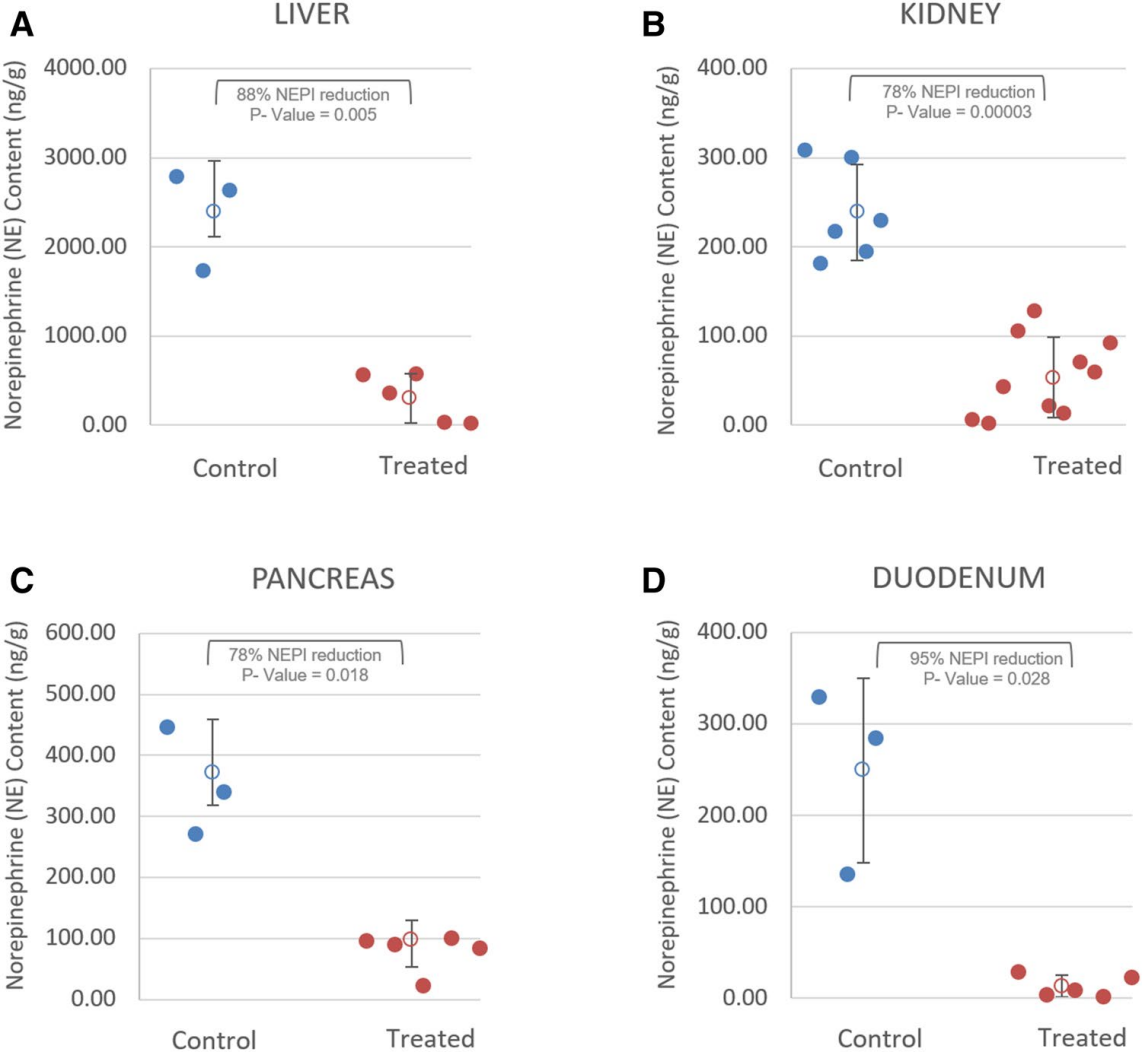
Tissue norepinephrine (NEPI) at 90 days in control and treatment animals. **a** Liver—points represent average NEPI of all five liver lobes from each animal. **b** Kidney—points represent individual kidney NEPI for each animal. **c** Pancreas—points represent average NEPI of head, body and tail of pancreas from each animal **d** Duodenum—points represent NEPI of the duodenual tissue associated with the pancreas from each animal. (blue circles: control animals; red circles: treated animals)

Norepinephrine concentrations were significantly reduced in the liver (*p* value = 0.005) and kidneys (*p* value = 0.00003) following multi-organ denervation treatment compared to control animals suggesting significant and sustained denervation of the organs through 90 days. Additionally, reductions in NEPI concentrations were noted in the pancreas and duodenal tissue suggesting partial denervation of these tissues surrounding the CHA. NEPI concentrations from all treatment and control animals are shown in Fig. 7a–d.

## Discussion

This study aimed to report the technical approach of a novel multi-electrode RF denervation system in a preclinical large animal model for multi-organ denervation and compare the safety and performance of the device against attributes ideal for clinical denervation.

To date, there are no reports in the literature regarding catheter-based denervation outside the kidneys, with only one publication on single organ ablation of the splenic artery [42]. In this study, catheter-based denervation in the CHA was shown to have no adverse effects on liver and pancreatic functional enzymes. Parameters related to bile production or secretion were not measured during this study. However, in other experimental animal models, hepatic denervation has shown to have no major effects on bile acid production [43]. Additionally, the safety of hepatic denervation is also supported by extensive experience with human liver transplantation, in which there is a complete disruption of innervation. Denervation following liver transplant has shown no evidence of abnormal liver function and has no significant deleterious effects on bile secretion, liver regeneration, or hepatic blood flow [43].

With this study, for the first time we determined the feasibility of a single procedure with a catheter-based technology, to simultaneously denervate multiple organs, including the liver, pancreas, duodenum and kidney. Using the annularity model created from fine histological sectioning and tracing, we uniquely showed that the effective lesion area created by a single iRF system treatment consistently ranged from 6–8 mm from the arterial lumen for both renal and CHA doses (Table 1), [44] which is deeper and more circumferential than those shown by other conventional RF devices [45]. In addition, the current study demonstrates that a denervation depth of of 6–8 mm from the arterial lumen can achieve a clinically significant reduction in NEPI levels in multiple organs by adequately denervating the perivascular space surrounding the renal arteries and CHA [36]. Similar renal cortical NEPI reductions have been reported in the literature with other RF devices through significantly increasing the number of RF lesions and lesion locations, both of which add to procedural burden to user and patient [20, 22, 25, 38]. The iRF Denervation System on the other hand, with its multi-electrode design produces a predictable circumferential lesion with a single treatment, removing the burden of accurate repositioning and creating multiple lesions.

The notable NEPI reduction observed in pancreatic and duodenal tissue was anticipated as both organs are supplied by the gastroduodenal artery branching off the CHA. It is well known that sympathetic neural activity to the pancreas inhibits insulin production and the reduction of sympathetic tone to the pancreas may be beneficial to improving insulin release [46]. Similarly, it has been suggested through experimental models that increased sympathetic tone to the intestine predominantly inhibits GLP-1 secretion and conversely, implies that lessening of the sympathetic intestinal tone could provide a stimulus for GLP-1 secretion [47]. Improving insulin and GLP-1 release could both have beneficial effects in glycaemic control [48].

The iRF Denervation System offers another distinction over conventional RF devices by incorporation of a cooling circuit that reduces the temperatures of the arterial wall that is in immediate contact with the balloon electrode assembly while allowing the thermal conduction of the RF energy to move through the adventitial space where the nerves reside. This is supported by no vessel abnormalities, narrowing or stenosis observed in any of the arteries treated with the iRF Denervation System and evident chronic nerve injury seen distant from the treated artery showing the conduction of RF energy in the adventitia.

The current study also demonstrated that the lesion can be deep enough to affect most of the nerves but not too deep to cause adverse retroperitoneal organ effects. This is supported by clinical observations and a thorough analysis of clinical pathology, that showed no clinically significant events in any animals treated with the iRF Denervation System through the follow-up. Histological observations such as focal pancreas and muscular necrosis were rare and minimal in this study and were considered clinically insignificant.

Extensive anatomical, preclinical, and clinical research on numerous RDN systems have been published over the past decade. The ablation depth of the currently available radiofrequency (RF)-based RDN systems ranges from 2 to 4 mm [49, 50], thereby potentially limiting the adequatee RF energy delivery in some sections of the renal artery. The authors assessed and reported the maximum average number of nerves observed in the proximal, middle and distal (pre-bifurcation) segments of the renal artery and the mean distance from the lumen to nerves in each segment. These data suggest that a target tissue treatment depth of ~ 6.4 mm will likely result in a 90th percentile nerve kills in renal arteries. Also, Mahfoud et al. reported the distribution of these nerves surrounding the renal arteries on a radial nerve distribution map, showing that 86% of renal nerves are within 6 mm in the main renal artery [51]. While there is no data published to date on catheter-based denervation of the CHA, the distribution of nerves around the CHA as well as the, microanatomical structures such as nearby vessels, organs and structures have been characterized using a human post-mortem histologic study (report on file with Metavention). These data indicate that > 95% of the nerves surrounding the CHA are efferentsympathetic nerves with 80% of the nerves localised within 6 mm from the CHA lumen. Moreover, an additional critical factor is that some nerves (e.g., renal) are surrounded by adipose tissue and lymph nodes [52] protecting them from the RF energy delivered by the conventional treatment approach. Thus, to perform effective arterial denervation, depth lesions fostered by a cooling system minimizing injury to the arterial wall and preventing lesions formation from being affected by anatomical heat sinks, such as surrounding veins, lymph nodes and other organs is necessary.

Although the location and distribution of renal sympathetic nerves seem relevant to achieve effective RDN, other factors may also be relevant. For instance, in 20 humans who underwent autopsy, renal sympathetic innervation was thoroughly assessed and found to vary widely in terms of nerve density and distribution [39, 51]. While the majority of the nerves were located in the proximal and middle segments of the arteries, the smallest number in the distal segments [39, 51]. Conversely, another human post-mortem histologic study showed that the number of nerves increased along the length of the artery from proximal to distal segments (proximal = 216; middle = 323; distal = 417) [41]. Also, data indicate that the nerves and ganglia are more plentiful at the proximal superior renal artery ostium [53]. However, high-frequency electrical renal nerve stimulation performed at multiple sites with a minimum of four locations in both arteries and ensuring that different quadrants of the arterial circumference were stimulated in proximal and distal areas of the renal artery, showed a heterogeneous response of the sympathetic nervous system [54, 55]. To achieve efficient denervation and to overcome issues with innervation distribution density and asymmetry along the artery, adequate depths and coverage of the entire circumference and length of the vessel is relevant [38]. Currently available RF technologies requier a large number of focal ablations in all accessible renal artery branches. It was reported that an average of 46.9 ± 15.6 ablations were performed in the SPYRAL HTN-OFF MED Pivotal trial to achieve adequate denervation [25]. It was also reported during this trial that only one operator per centre was allowed to perform the RDN procedures to minimize user variability, which may represent a limitation for devices that require large number of ablations for success [25].

In summary, in this study, we demonstrated the ability of the iRF Denervation System to create a deep predictable circumferential lesion which translated to significant reduction in NEPI levels in multiple organs, i.e. liver, pancreas, duodenum and kidney. The incorporation of a cooling balloon and the ability of the system to target nerves surrounding both the renal arteries and CHA at varying depths without compromising vascular integrity or creating clinical adverse events suggest that the technology is versatile as well as safe. The iRF Denervation System design advances RF denervation technologies in the right direction by demonstrating the ability to meet the ideal characteristics for denervation which could translate to several procedural benefits and ease of use in a clinical setting.

### Limitations

Preclinical studies are the primary means of evaluating treatment efficacy prior to first in human studies. However, limitations may apply to clinical translation of preclinical findings such as potential tissue and anatomical differences, and histomorphology measurements commonly affected by tissue shrinkage underestimating treatment effect. Physiologic parameters for blood pressure and glycaemic control (HbA1c, insulin, GLP-1) were not monitored in this healthy swine model and, therefore, clinical validation in patients with hypertension and T2DM is needed.

## Conclusions

The results of this multi-organ study, support the feasibility of a novel iRF Denervation System to effect denervation in the multiple organs, i.e. liver, pancreas, duodenum and kidney from a single denervation procedure to bilateral renal arteries and CHA as shown through reduction is tissue NEPI. This device has unique advantages over conventional RF devices including ability to maintain arterial integrity through active cooling which was verified through no stenosis seen in follow-up angiography. The iRF Denervation System used in renal arteries and CHA appear to be safe for human use as supported by angiography, clinical pathology, gross and microscopic pathology assessments. Clinical trials are underway to confirm the favourable efficacy and safety profile of the iRF Denervation System in patients with hypertension and T2DM.
